# Cocaine

**Published:** 1888-03

**Authors:** Frank Trester Smith

**Affiliations:** Chattanooga, Tennessee, Late Attending Surgeon Eastern Dispensary, Department Eye, Ear and Throat New York


### COCAINE.

---

BY FRANK TRESTER SMITH, M. D., CHATTANOOGA, TENNESSEE,

Late Attending Surgeon Eastern Dispensary, Department Eye, Ear and Throat New York.

---

About two years ago the editor of the New York *Medical Record* said that the most lonesome man in the world was the ophthalmologist who had not written an article on cocaine. It was in connection with ophthalmic surgery that it was introduced to the profession; for, although it was known long before Köller's discovery, and had been used to produce local anæsthesia of the pharynx and larynx, both in this country and in Europe, its usefulness was not appreciated.

It is to the oculists that the profession is indebted for the introduction of this agent. As soon as they came to know its value in eye surgery, they carried their experiments to other regions of the body, the results of which were at once given the profession.

So much has been written that it is almost impossible to add anything new; it will be the endeavor to emphasize some points that may have been forgotten, and to give an item or two that will be new to some.

Cocaine is an alkaloid abstracted from the leaf of the erythroxylon coca. It crystallizes in colorless prisms. It forms salts with acids, of which the following are in use:

Cocaine oleate, cocaine citrate, cocaine hydrobromate, cocaine salicylate, cocaine hydrochlorate or muriate.

The last named is the one in general use, being preferred to the alkaloid on account of its solubility. The solubility of the alkaloid is variously given as one in 704 parts of cold water to one in 2,000, while the muriate is readily soluble. Generally speaking, when the term cocaine is used, the hydrochlorate is referred to.

The most practicable test of its purity lies in the odor, that preparation having the least odor being freest from impurities.

The preparations of this salt, as found in the stores, differ somewhat. Most often it comes as an amorphous powder. Squibbs' is such. Schieffelin's come in small crystals. Parke, Davis & Co. have large and small crystals. When a cocaine solution is dropped into the eye the first sensation is one of smarting. This fact has escaped the notice of most writers on this subject. Some patients complain very bitterly of this. The pain is in direct ratio to the strength of the solution. The large crystals before alluded to are freest from this irritating principle which Parke, Davis & Co. say may be due to higrine, a substance found in the coca leaf and which manufacturers have been unable so far to eliminate in preparing cocaine. It is partly gotten rid of by repeated crystallizations.

Higrine gives off the odor of bitter almonds, and the amount of the odor is a ready test of the amount of higrine in a given solution. This substance has never been isolated, so that we know positively very little about it.

The smarting above referred to is not noticed when solutions are used on the nasal mucous membrane or on other parts of the body. Preparations other than the large crystals are to be preferred here, as they are somewhat cheaper.

With the use of cocaine about the eye all are familiar. It has been employed for every operation from the removal of a foreign body to enucleation of the globe.

Attention may be called to one or two points: First, the strength of the solution. Experiments with a 20 per cent. solution in operating give no better results than a 4 per cent. solution. The deep parts, iris, etc., may be anæsthetized by repeated instillations every five minutes for twenty or thirty minutes.

In connection with instillations in the eye, something may be said of what is known as the "cocaine accident." Dr. Knapp first called attention to this. The phenomena are about as follows: The face becomes pale and sometimes covered with a clammy sweat. The pulse is weak and very rapid. Loss of consciousness may or may not supervene.

Dr. J. L. Minor, of Memphis, points out that the symptoms are those of an ordinary fainting fit, and attributes all of the phenomena to the mental impression produced by the knowledge that an operation is being performed.

This explanation may suffice for some cases, but it should not be forgotten that there is a case on record where death resulted from the introduction of three grains into the rectum.

The accident is comparatively rare, occurring in less than one-half per cent. of cases, speaking without statistics. It has occurred to me four times, so far as I remember, the most marked case being the following:

Shortly after the introduction of cocaine, while operating for squint, in a tenement house in New York, the patient being a boy ten years old, it was noticed that he became quite pale; the face was covered with sweat; he asked for a drink of water and almost immediately lost consciousness. Thinking that he had fainted, he was placed in a horizontal position, some brandy forced between his teeth and the operation completed. By the time the eye was bandaged, he had fully recovered.

Cocaine is generally described as a heart tonic, so that these must be regarded as idiosyncrasies.

Dr. Calhoun, of Atlanta, in enucleating an eye-ball, using a ten per cent. solution, gives the patient a dram of whisky as a precaution. He says this will counteract any depressing effect of the cocaine. He then injects five minims over the internal rectus muscle and the same amount over the external rectus.

The cocaine here has the double effect of producing anæsthesia and lessening the loss of blood.

The oculist finds another use for cocaine in the quality it possesses of dilating the pupil. On account of its very slight action of the muscle of accommodation, it is to be preferred to atropine. The other mydriatics are not as often used on account of their cost—the price of homatropine, for instance, being seventy-five cents a grain.

Cocaine is often of great service where it is desired to make an examination of the parts of the eye behind the iris without subjecting the patient to the inconvenience of paresis of the accommodation.

Cocaine is not absorbed through the unbroken skin. Most authorities claim that it is of little use for pain in the ear unless there is a perforation, but this is a mistake; the relief from a four per cent. solution warmed is often considerable.

For aural polypi it is clearly indicated, rendering their removal by instrumental means much more easy.

In nasal diseases cocaine has a wide range of application, being used for the following purposes:

1. To produce anæsthesia.

2. To reduce turgercense.

This is a valuable property greatly facilitating examinations, assisting in the removal of foreign bodies, and rendering more easy other operative maneuvers.

3. To lessen secretion.

In acute coryza, as soon as the secretion begins to flow, a two or four per cent. solution may be sprayed into the anterior nares. It will entirely suppress the secretion. Its effects last about three-quarters of an hour. It is a valuable aid in aborting a cold in the head, an important matter in the treatment of nasal catarrh.

For removing foreign bodies and for operations, a twenty per cent. solution is to be preferred for two reasons: 1st. The result is accomplished in a much shorter time. 2nd. Less of the drug is required to produce the desired effect.

It is best applied with a pledget of cotton wound on the end of a probe, or it may be saturated with the solution and left in *situ*.

In the use of this drug in any part of the body, the precaution should be observed to use less than three grains, that amount having produced death in one case. There is a case on record where twenty grains were swallowed, but there is some doubt of the purity of the preparation.

In minor surgery, the drug must be introduced under the skin. There are two methods of doing this: first, by the hypodermic syringe. The objection to this is the pain produced by the puncture. To obviate this, and to prolong the anæsthesia, Dr. J. Leonard Corning, of New York, devised the following method :

The skin is first anæsthetized by means of a spray of ether, or better, rhigolene. The cocaine is then introduced by means of the hypodermic syringe. Now the blood-current will soon carry away the solution unless it is retarded. For this purpose, a ring an inch and a half in diameter, serving to support a dome of fine wire gauze, is attached to a handle by means of a bifurcation. In using the instrument, the cutaneous circulation is obstructed by the wires, which are pressed tightly upon the part to be anæsthetized, while the meshes between the wires allow the spray to come in contact with the skin. Other means of retarding the circulation, as the Esmarch bandage, or the ordinary roller bandage, are sometimes employed.

This method of producing local anæsthesia deserves more attention than it has received from the profession. For minor operations, and for the treatment of painful affections, it is eminently useful. It is claimed that obstinate neuralgias have thus been cured.

Internally cocaine is not used to any great extent. It is recommended to obviate the sense of fatigue from prolonged labor, but this practice is not justifiable. The formation of a cocaine habit is not altogether visionary.

